# Post ablation timing to best visualize left-atrial Llesions: a feasibility study

**DOI:** 10.1186/1532-429X-18-S1-P203

**Published:** 2016-01-27

**Authors:** Ibrahim Saeed, Joseph S Soltys, Sanjaya Gupta, Ryan Longmore

**Affiliations:** 1 Cardiovascular Imaging Technologies, Kansas City, MO USA; 2 Saint Luke's Mid America Heart Institute Cardiovascular Consultants, Kansas City, MO USA; 3 University of Missouri - Kansas City, Kansas City, MO USA

## Background

Catheter-based atrial fibrillation (AF) therapy often uses cryo-balloon ablation of pulmonary vein (PV) ostia to achieve rhythm control. Prior studies suggest the ability of late gadolinium enhancement cardiac MRI (LGE-MRI) to visualize radiofrequency-induced scar. The optimal time after therapy to visualize cryo-ablation lesions is unknown. This study evaluates the relationship between the time of LGE-MRI acquisition after ablation and visualization of cryoballoon-induced scar.

## Methods

Eleven consecutive patients (9 M, age 61 ± 6y) undergoing cryoablation for AF were prospectively enrolled. All patients had pre-procedural left atrial angiography with LGE-MRI (1.5T Signa HDx, General Electric, ) to define left atrial and pulmonary vein (PV) anatomy. All patients underwent repeat LGE-MRI between 7-28 days post-ablation. Images were visually assessed for scar (estimated as percent circumferential enhancement) detected in the PV antrum and placed in quartiles of definite LGE, likely LGE, unlikely LGE, and no LGE.

## Results

44 PVs were assessed both pre- and post-procedure, and all but 6 pre-procedural were characterizable. At < 14 days post-procedure, there was 31.8% definitely LGE circumferentially vs. 49.3% LGE > 20 days (p= 0.053); and a combined definite and likely LGE of 59.7% circumferential LGE vs 72.9% > 20 days (p = 0.002), despite achieving electrical PV isolation in all 44 patients.

## Conclusions

This study demonstrates the feasibility of observing cryoablation induced PV scar on LGE-MRI by delaying imaging until 20 days post-ablation.

**Figure 1:**
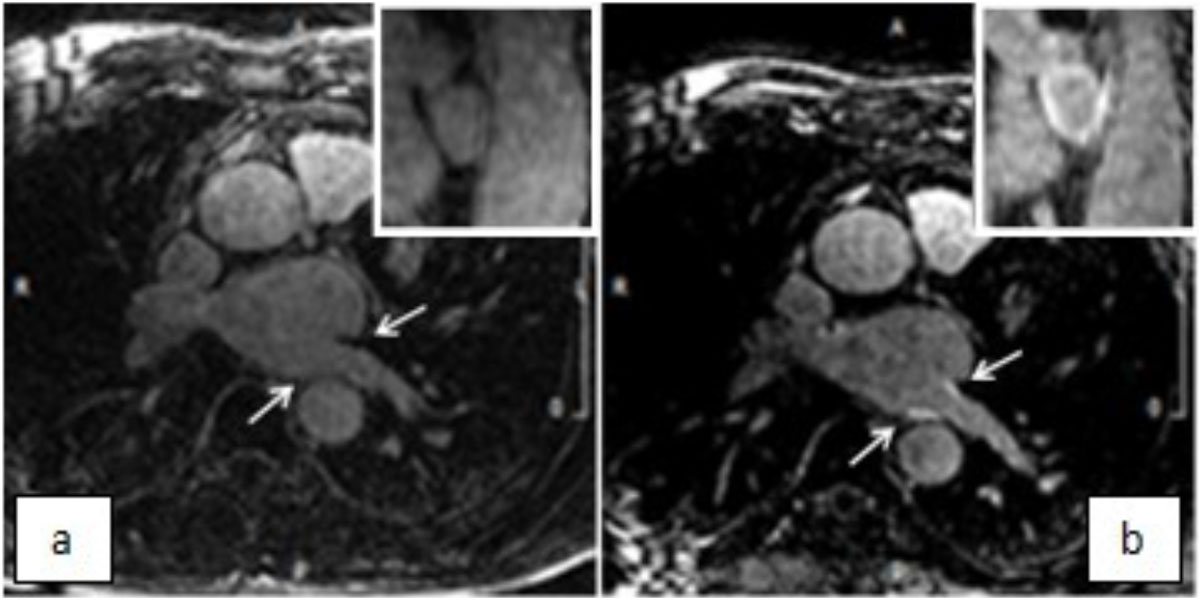
**Typical acquisition showing pre ablation (a) and 20 days post ablation (b) LGE**.

**Figure 2:**
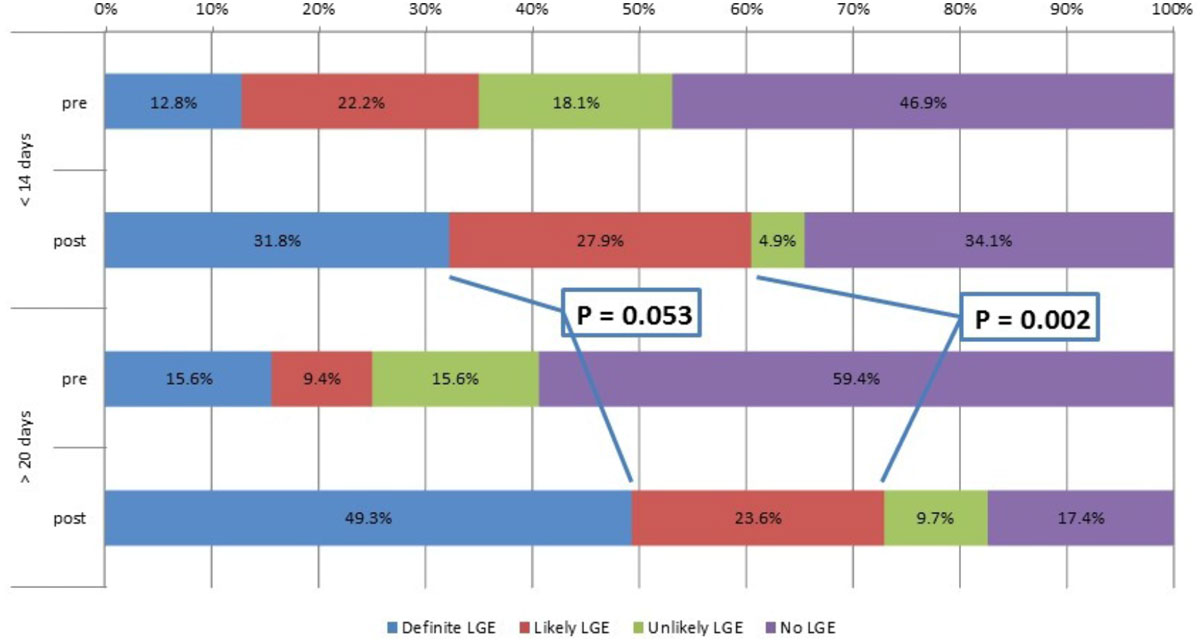
**Quartiles of pulmonary vein circumferential LGE pre-, less than 14 days post-, and greater than 20 days post-ablation**.

